# mSphere of Influence: Missing the trees for the forest

**DOI:** 10.1128/msphere.00737-25

**Published:** 2026-02-12

**Authors:** Katherine Rhodes

**Affiliations:** 1 Immunobiology Department, University of Arizona College of Medicine-Tucson 12216, Tucson, Arizona, USA; 2 BIO5 Institute, University of Arizona, Tucson, Arizona, USA; The University of Arizona, Tucson, Arizona, USA

**Keywords:** commensalism, microbe-microbe interaction, inter-bacterial antagonism

## Abstract

Katherine Rhodes is a bacteriologist working in the field of host-microbe interaction. In this mSphere of Influence article, she reflects on how two papers, “Spatial ecology of the human tongue dorsum microbiome” by S. Wilbert, J. Mark Welch, and G. Borisy and “Novel peptide from commensal *Staphylococcus simulans* blocks methicillin-resistant *Staphylococcus aureus* quorum sensing and protects host skin from damage” by M. Brown et al., impact her research on *Neisseria* commensalism and host adaptation.

## COMMENTARY

Our understanding of the human ecosystem and its many microbial inhabitants has grown by leaps and bounds since Falkow’s 1988 molecular Koch’s postulates (1). Over time, this mechanistic framework for host-microbe interaction has yielded seminal insights into microbial behavior, aided in part by technological advances which allowed study of these interactions in increasingly fine detail. What is most interesting to me, however, is the application of these approaches not only to the study of pathogenesis but also to commensal species, which often garner less attention at a molecular scale. These organisms must be consummate survivors as components of the microbiota in many hosts. They have evolved the tools to maintain the delicate balance of host manipulation, inter-species antagonism, and metabolic adaptation that secures their niche. However, the study of commensal biology at a mechanistic level still lags behind pathogenesis despite the value of understanding how “the microbiota” functions, not just its bulk composition.

The development of 16s amplicon sequencing revealed the makeup of microbiota communities, but could not provide information on their organization, a critical component of niche ecology. Now, new technologies allow analysis of these communities in increasingly physiologically relevant contexts. In “Spatial ecology of the human tongue dorsum microbiome,” the authors use the novel microscopy technique, combinatorial labeling and spectral imaging-fluorescence *in situ* hybridization (CLASI-FISH), to determine the structure of microbial consortia in the human mouth (2). Tongue scrapings were harvested from volunteers and processed for imaging. Using multiple FISH probes per taxon, each labeled with different fluorophores, and a laser scanning confocal microscope with a spectral detector, the authors determined both the identity of the organisms within these preserved, naturally occurring biofilms and the position of each organism within its environment for the first time. This offered a high-resolution depiction of the organization of these complex communities. The authors determined how these consortia were structured and which organisms co-localized within each microenvironment, providing evidence for the existence of site-specialist phenotypes. Combining this approach with metagenomic sequence analysis further described the gene content of these communities and offers insight into the potential mechanisms used to support survival within these microenvironments.

The exploration of commensal biology shows great promise not only from a basic science view but from a translational perspective as well. In their 2020 report “Novel peptide from commensal *Staphylococcus simulans* blocks methicillin-resistant *Staphylococcus aureus* quorum sensing and protects host skin from damage,” the authors dissect how inter-species antagonism could contribute to improved host health (3). The paper focused on interactions between coagulase-negative *Staphylococcus* species and *S. aureus*. In *S. aureus*, expression of the quorum sensing accessory gene regulator (*agr*) system facilitates expression of the RNAIII virulence regulon. However, the *agr* system is conserved across the genus, including in many *Staphylococcus* species associated with *S. aureus* colonization resistance. Building on this observation, the authors interrogated a panel of commensal staphylococci for their ability to modulate expression of non-cognate *agr* loci using multiple reporter strains. The authors observed substantial inter- and intra-species cross talk between different variants of the autoinducing peptide (AIP) recognized by the *agr* system histidine kinase. These assays showed particularly potent inhibition of a methicillin-resistant *S. aureus*-derived *agr-I* by the AIP of *S. simulans*, prompting further evaluation of this AIP as a potential anti-infective. Mice inoculated with *S. simulans* concurrent to *S. aureus* infection or treated with synthetic *S. simulans* AIP-I both showed a marked reduction in *S. aureus*-related pathologies. Given the rapid rise of antimicrobial resistance in many pathogens, studies like this one highlight the merit of a closer inspection of commensal biology to identify potential tools in a therapeutic arsenal.

These reports set an encouraging precedent for my own group's research on the commensal *Neisseria*. Most prior work has focused on *Neisseria* pathogenesis rather than homeostasis within the mucosal niche. In humans, commensal *Neisseria* are an abundant taxon of the oral cavity, coexisting with numerous other organisms while maintaining a (mostly) non-antagonistic relationship with their host. These microbe-microbe interactions were directly captured *in situ* by Wilbert et al., offering us insight into the identity of commensal *Neisseria’s* nearest neighbors. Moreover, like in *S. simulans*, evaluation of the basic mechanisms which drive commensal *Neisseria* behavior may hold translational promise. Recent reports on the mechanisms of interbacterial antagonism between *Neisseria* suggest the possibility that these processes can be employed for our own benefit (4–6). Our hope is that continued progress delving into commensal biology will help us fully understand the impact of the microbiota on human health and disease.
